# Occupational gaps 5 years after stroke

**DOI:** 10.1002/brb3.1234

**Published:** 2019-02-19

**Authors:** Joel S. Svensson, Emma Westerlind, Hanna C. Persson, Katharina S. Sunnerhagen

**Affiliations:** ^1^ Department of Clinical Neuroscience Institute of Neuroscience and Physiology Sahlgrenska Academy University of Gothenburg Gothenburg Sweden

**Keywords:** follow‐up, occupational gaps, participation, stroke

## Abstract

**Objectives:**

To investigate the incidence and number of occupational gaps 5 years after stroke and find possible predictors and explanatory factors for increased number of experienced gaps.

**Material and Methods:**

The participants were diagnosed with first‐time stroke in Gothenburg during 2009–2010. Medical records from their hospital stay were used to obtain baseline data. The Occupational Gaps Questionnaire and the Swedish stroke registers follow‐up questionnaire were sent out. Data from the Occupational Gaps Questionnaire were used as a dependent variable and baseline data and questions from the stroke registry were used as independent variables in logistic regression.

**Results:**

Five years poststroke, 49.5% experienced a higher number of occupational gaps compared to a healthy reference population. Predictors for an increased number of gaps were higher age at stroke onset and a higher degree of functional dependency. Explanatory factors for an increased number of gaps in the study population were higher age at follow‐up and feelings of depression.

**Conclusions:**

Older age at the time of stroke and functional dependency can predict an increased number of occupational gaps. Older age and feelings of depression are connected to an increased number of occupational gaps. Individuals at risk should be provided with additional interventions to reduce participation restrictions.

## 1 INTRODUCTION

Stroke is a common disorder that can result in a wide array of impairments, both physical and cognitive. These impairments can severely limit an individual's ability to carry out daily activities, resulting in reduced participation.

Participation is often seen as a central concept and a main goal in rehabilitation (Lecture, 2005) and is something that occurs at the intersection of what a person can do, wants to do, has the opportunity to do, and is not prevented from doing (Mallinson & Hammel, 2010). Participation can be seen from both an objective and a subjective point of view. While the objective aspect focuses on observable behaviors and activities, the subjective targets the individual's own perception of participation (Dijkers, 2010) and is important since it is more connected to the individual's own goals and priorities rather than the actual disability (Witsø, Eide, & Vik, 2012) and thus, can affect the course and result of rehabilitation. This highlights the value of obtaining the individual's own view and thoughts on their situation rather than only observing and quantifying their abilities and limitations.

Approximately two thirds of people experience reduced participation 6 years after stroke compared to prestroke (Singam, Ytterberg, Tham, & von Koch, 2015; Spitzer, Tse, Baum, & Carey, 2011). These high numbers and the connection between participation and life satisfaction (Cooper et al., 2014; Eriksson, Kottorp, Borg, & Tham, 2009) makes reduced participation after stroke an important aspect of rehabilitation. A number of studies have investigated the level of participation after stroke (Desrosiers et al., 2006; Spitzer et al., 2011; van der Zee, Visser‐Meily, Lindeman, Jaap Kappelle, & Post, 2013; Vincent‐Onabajo & Blasu, 2016) but only a few in a longer time‐period than 4 years poststroke (Bergström, Guidetti, Tham, & Eriksson, 2017; Singam et al., 2015). Several factors, such as age, physical dependency, and comorbidity have been identified as predictors for reduced participation at different time points following a stroke (Desrosiers et al., 2006; Singam et al., 2015; van der Zee et al., 2013). Though some factors seem to be reoccurring as predictors for reduced participation, these studies present varying results, leading to an inconclusive view of the situation. Thus, further research is warranted to identify and solidify which factors contribute to reduced participation following a stroke.

The Occupational Gaps Questionnaire assesses participation through restrictions in occupational activities (Eriksson, Tham, & Kottorp, 2013) and has been used in several studies to investigate occupational gaps after stroke and predictors for an increased number of occupational gaps after stroke (Bergström et al., 2017; Bergstrom et al., 2012; Eriksson, Tham, & Borg, 2006). These studies have shown a wide extent of occupational gaps after stroke with up to 90% of a study population experiencing occupational gaps (Bergstrom et al., 2012), making it an interesting and important aspect to further explore.

The aim of the study was to investigate the incidence and number of occupational gaps 5 years after stroke and find possible predictors and explanatory factors for increased number of experienced gaps.

## 2 MATERIAL AND METHODS

The present study is based on data collected in the SALGOT extended study. Participants were recruited for the SALGOT extended study at Sahlgrenska University Hospital/Sahlgrenska between February 4, 2009 and December 2, 2010. The SALGOT extended study comprises of three different populations treated for first time stroke at a stroke unit, a neurosurgical unit, and an intensive care unit (Persson, Parziali, Danielsson, & Sunnerhagen, 2012; Vikholmen, Persson, & Sunnerhagen, 2015; Wesali, Persson, Cederin, & Sunnerhagen, 2015).

Inclusion criteria for the SALGOT‐extended study were the following. Being diagnosed with first time stroke (defined as ischemic stroke I63, intracerebral hemorrhage I61, and subarachnoid hemorrhage I60) and being 18 years or older as well as living within 35 km of Sahlgrenska University Hospital.

**Figure 1:**
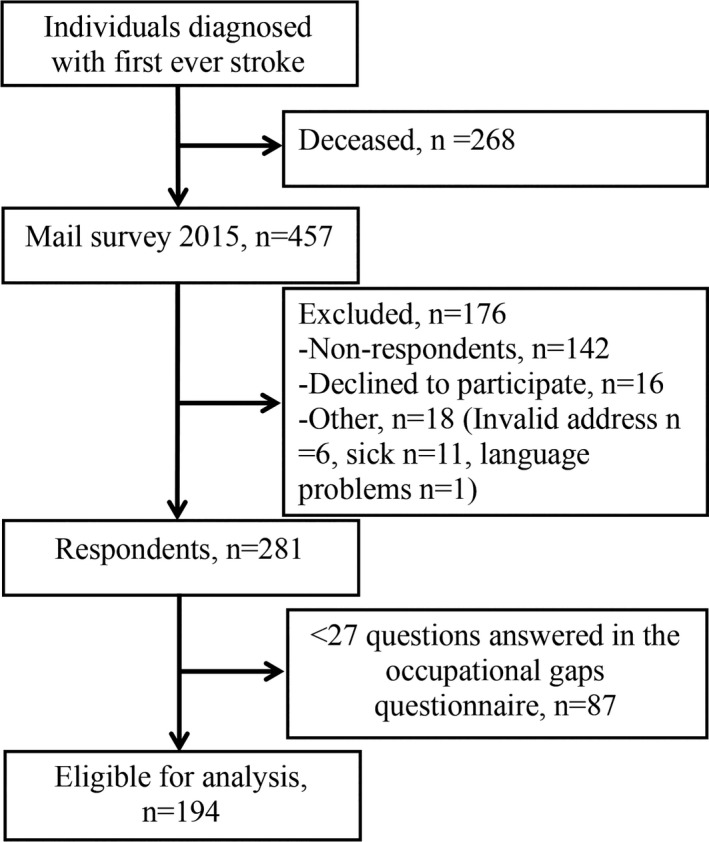
Flow chart of the study population

As seen in Figure 1 the SALGOT extended study included 725 individuals. A set of questionnaires was sent out 5 years later to the living participants (*n* = 457). Out of these participants, 281 completed the survey. Participants with incomplete answers in the occupational gaps questionnaire (<27 out of 30 questions answered) were also excluded, resulting in 194 individuals eligible for analysis.

### 2.1 Questionnaires

The Occupational Gaps Questionnaire (Eriksson, 2012) measures perceived occupational gaps. The questionnaire presents 30 different questions covering work, leisure, and social activities. For each of the activities, two questions are answered with yes or no: (a) *Do you perform the activity?* and (b) *Do you want to perform the activity?* Any discrepancy in the answers to the questions is identified as an occupational gap. The gaps can be divided into: (a) the gaps when a person wants to do an activity but is not able to (WTD gaps); and (b) the gaps when a person does an activity he/she does not want to do (DNWTD gaps). A lower number of occupational gaps is seen as a better outcome. The scale has been shown valid in different patient populations in Sweden.

The Swedish stroke register's follow‐up questionnaire consists of a number of questions regarding patients’ health, capacity in different activities, and feeling of support following their stroke. For this study, the questions regarding feelings of depression, living alone, re‐stroke, and feeling of support were used.

### 2.2 Stroke measurements

Clinical data were gathered from patient records. The National Institutes of Health Stroke Scale (NIHSS) (Lyden et al., 1999) was used to assess stroke severity at arrival to hospital of patients with ischemic or hemorrhagic stroke. For patients with subarachnoid hemorrhage, the Hunt & Hess scale (H&H) (Hunt & Hess, 1968) was used at admission instead. The modified Rankin Scale (mRS) (van Swieten, Koudstaal, Visser, Schouten, & van Gijn, 1988) was used to assess functional dependency at discharge for all patients.

The NIHSS is a scale that is used to assess neurological function in patients with stroke, ranging from 0 to 46 points where a lower score equals fewer stroke symptoms. For this study the following cut‐off values are used; very mild (0–2), mild (2–4), moderate (5–15), and severe (16–46) stroke.

The H&H scale is used on patients with SAH and the five stages of the scale represent the following states; I = asymptomatic, II = moderate to severe headache without neurological deficits, III = reduced consciousness with or without focal symptoms, IV = unconsciousness with or without focal symptoms, V = deep coma.

Stroke severity: To investigate stroke severity in participants with ischemic or hemorrhagic stroke as well as those with SAH, a combined variable for NIHSS and H&H was created based on clinical experience, and later dichotomized. Very mild to mild stroke according to NIHSS corresponds to grade 1 and 2 in H&H, and moderate to severe stroke according to NIHSS corresponds to grade 3–5 in H&H.

The mRS is a scale used to measure functional independence. The scale ranges from 0, indicating total functional independence, to 5, indicating severe disability, with 6 indicating death. This scale has a proven validity and reliability in the context of stroke in a clinical setting. A score of ≥3 is considered poor outcome in this study.

### 2.3 Statistical methods

The SPSS (version 25) was used to carry out statistical analyses.

Logistic regression was used to determine potential predictors and explanatory variables. The study population was divided into two groups according to the number of occupational gaps compared to an age matched reference population of 811 persons living in Sweden (Eriksson, 2012). One group with more than the median number of gaps and one group with the same or lower number of gaps as the median in respective age group. The median number of gaps in the reference population were as follows; age 20–29 = 5 gaps, age 30–49 = 4 gaps, age 50–64 = 2 gaps, age >65 = 1 gap (Eriksson, 2012). Having more gaps than the reference population was used as the dependent variable in logistic regression. Age, functional dependency (mRS), stroke severity, sex, feelings of depression, feeling of support, living alone, and re‐stroke were used as independent variables.

Functional dependency (mRS), stroke severity, feelings of depression, and feeling of support were dichotomized based on clinical relevance and to ensure sufficient group sizes. The cut off used for dichotomization of functional dependency (mRS) was a score of 3, where scores <3 were considered a favorable outcome and scores ≥3 were considered a poor outcome. Feelings of depression were dichotomized by creating one group who reported that they never, almost never, or sometimes felt depressed, the other group reported having feelings of depression often or always. Dichotomization of the variable feeling of support was done by creating one group who reported no need for additional support and one group who reported insufficient feelings of support.

Cross tabulations were used to ensure large enough groups (*n* ≥ 5) for each variable. Correlations between variables were explored using Spearman's rank order, with values of ±≤0.7 deemed eligible for further analysis. Univariate analysis was carried out on all independent variables and variables with a *p*‐value ≥0.25 were excluded. The remaining variables were used in a multivariable logistic regression. As a final step, the excluded variables were put into the final models to check for significance.

Reliability of the models was established with the Hosmer and Lemeshow test with a *p*‐value of >0.05 indicating reliability. Cox and Snell and Nagelkerke R square investigate the variation in the outcome explained by the regression model. A ROC‐curve was performed to give an indication of the model's accuracy and an area under the curve of >0.7 was considered acceptable.

### 2.4 Ethics

The SALGOT study was approved by the Regional Ethics Committee in Gothenburg (EPN) in 2008 (Dnr: 225‐08 and T801‐10) and followed the Helsinki declaration. Since the data acquired from medical records were used for clinical purposes and quality control, informed consent was not required (personuppgiftslagen, Swedish law No. SFS 1998:2014). The follow‐up survey was approved in June 2013 (Dnr: 400‐13).

## 3 RESULTS

The characteristics of the study population at the time of stroke are shown in Table 1. The mean age of the individuals included at the time of stroke was 63.0 years (*SD* 14). Most participants were male (62.4%). 73.7% of the included individuals suffered an ischemic stroke. Most participants suffered from a very mild to mild stroke (71.6%).

**Table 1:** Baseline characteristics of the study population

Age	
Mean (*SD*)	63.0 (14)
Median (min‐max)	64 (24–90)
Gender, *n* (%)	
Male	121 (62.4)
Female	73 (37.6)
Type of stroke, *n* (%)	
IS	143 (73.7)
ICH	27 (13.9)
SAH	24 (12.3)
NIHSS score at admission, *n* (%) Total *n*=154	
Very mild 0–2	96 (62.7)
Mild 2–4	15 (9.8)
Moderate 5–15	28 (18.3)
Severe 16–46	14 (9.2)
Mean (*SD*)	4.0 (5.8)
Median (min‐max)	1 (0–24)
MRS at discharge, *n* (%)	
0	3 (1.5)
1	26 (13.4)
2	64 (33.0)
3	54 (27.8)
4	40 (20.6)
5	7 (3.6)
H&H, *n* (%) Total *n* = 22	
Grade 1	2 (9,1)
Grade 2	12 (54.5)
Grade 3	2 (9.1)
Grade 4	5 (22.7)
Grade 5	1 (4.5)
Stroke severity, *n* (%) Total *n* = 176	
Very mild to mild (NIHSS), Grade 1–2 (H&H)	126 (71.6)
Moderate to severe (NIHSS), Grade 3–5 (H&H)	50 (28.4)
Stroke location, *n* (%) Total *n* = 173	
Left hemisphere	92 (53.2)
Right hemisphere	64 (37.0)
Bilateral	7 (4.0)
Cerebellum	4 (2.3)
Unknown	6 (3.5)

IS: ischemic stroke; ICH: intra cerebral hemorrhage; SAH: sub arachnoidal hemorrhage; NIHSS National Institute of Health's Stroke Scale; mRS: modified Rankin Scale, H&H: Hunt and Hess.

At time of follow‐up, the mean age was 69.8 years. Thirty participants (15.5%) suffered a new stroke during the time between study inclusion and follow‐up. 13.9% reported having feelings of depression often or always.

The nonrespondents were significantly more females (*p* = 0.001) and more moderate–severe stroke cases (*p* = 0.023) compared to respondents. Individuals with an incomplete (<27 questions answered) OGQ were significantly older (*p* = <0.001) compared to individuals with a complete OGQ.

**Figure 2:**
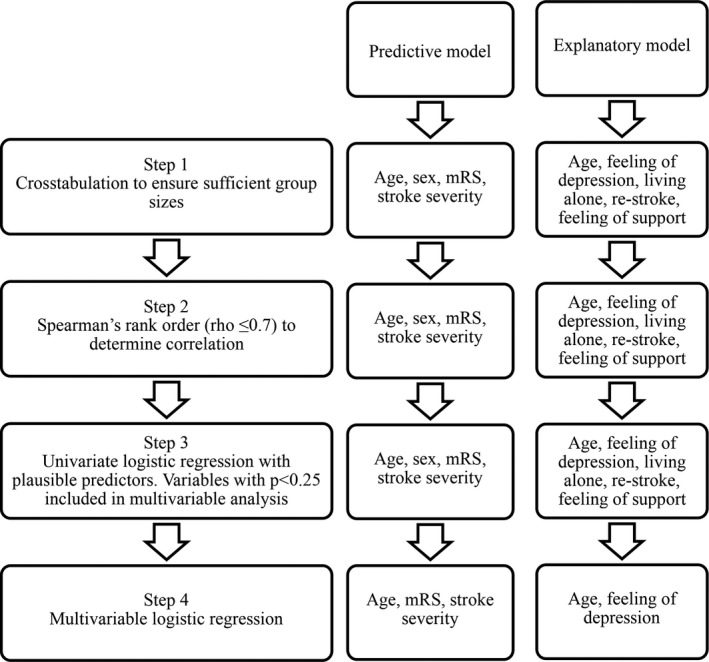
Different types of gaps for each activity in the Occupational Gaps Questionnaire (WTDG: want to do gaps; DNWTDG: do not want to do gaps)

**Figure 3:**
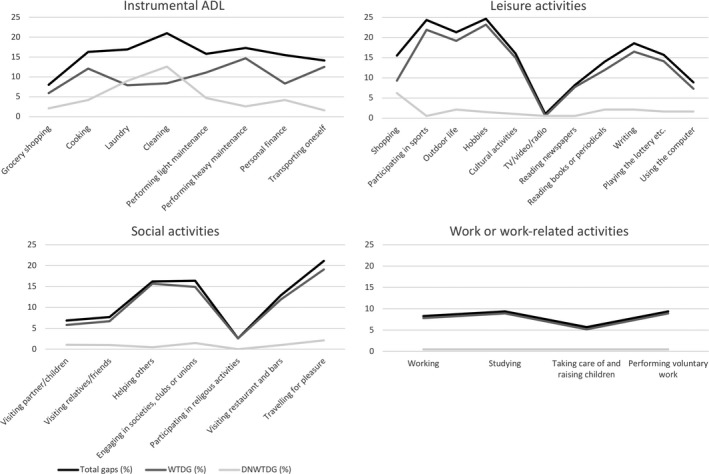
Illustration of the model building in the two logistic regression models (mRS: modified Rankin Scale)

The number of perceived gaps in the different activities of the OGQ is displayed in Table 2. Most of the included individuals (70.6%) perceived occupational gaps 5 years after stroke (Figure 2). The median number of occupational gaps was 2 (range 0–22). The activity *hobbies* showed the highest percentage of individuals reporting occupational gaps with 24.7%. The activity *cleaning* showed the highest percentage of DNWTD gaps (12.6%). A total of 96 (49.5%) individuals experienced more occupational gaps than the median in the reference population. The process to identify predictive and explanatory factors for increased occupational gaps 5 years after stroke is shown in Figure 3.

**Table 2:** Number and types of occupational gaps in each activity in the Occupational Gaps Questionnaire

Activity	Total *n*	Total gaps *n* (%)	WTD gaps *n* (%)	DNWTD gaps *n* (%)
Instrumental ADL				
Grocery shopping	188	15 (8.0)	11 (5.9)	4 (2.1)
Cooking	190	31 (16.3)	23 (12.1)	8 (4.2)
Laundry	189	32 (16.9)	15 (7.9)	17 (9.0)
Cleaning	190	40 (21.0)	16 (8.4)	24 (12.6)
Performing light maintenance	190	30 (15.8)	21 (11.1)	9 (4.7)
Performing heavy maintenance	190	33 (17.3)	28 (14.7)	5 (2.6)
Personal finance	192	24 (15.5)	16 (8.3)	8 (4.2)
Transporting oneself	192	27 (14.1)	24 (12.5)	3 (1.6)
Leisure activities				
Shopping	194	30 (15.5)	18 (9.3)	12 (6.2)
Participating in sports	192	43 (24.4)	42 (21.9)	1 (0.5)
Outdoor life	193	41 (21.3)	37 (19.2)	4 (2.1)
Hobbies	194	48 (24.7)	45 (23.2)	3 (1.5)
Cultural activities	193	31 (16.0)	29 (15.0)	2 (1.0)
TV/video/radio	194	2 (1.0)	1 (0.5)	1 (0.5)
Reading newspapers	194	16 (8.2)	15 (7.7)	1 (0.5)
Reading books or periodicals	193	27 (14.0)	23 (11.9)	4 (2.1)
Writing	194	36 (18.6)	32 (16.5)	4 (2.1)
Playing the lottery etc.	191	30 (15.7)	27 (14,1)	3 (1.6)
Using the computer	193	17 (8.9)	14 (7.3)	3 (1.6)
Social activities				
Visiting partner/children	190	13 (6.9)	11 (5.8)	2 (1.1)
Visiting relatives/friends	194	15 (7.7)	13 (6.7)	2 (1.0)
Helping others	191	31 (16.2)	30 (15.7)	1 (0.5)
Engaging in societies, clubs, or unions	194	32 (16.4)	29 (14.9)	3 (1.5)
Participating in religious activities	191	5 (2.6)	5 (2.6)	0 (0)
Visiting restaurants and bars	193	25 (12.9)	23 (11.9)	2 (1.0)
Travelling for pleasure	194	41 (21.1)	37 (19.1)	4 (2.1)
Work or work‐related activities				
Working	193	16 (8.3)	15 (7.8)	1 (0.5)
Studying	191	18 (9.4)	17 (8.9)	1 (0.5)
Taking care of and raising children	191	11 (5.7)	10 (5.2)	1 (0.5)
Performing voluntary work	192	18 (9.4)	17 (8.9)	1 (0.5)

WTD: want to do; DNWTD: do not want to do.

The results from the predictive model showed that a higher age at stroke onset (OR = 1.045 95% C.I. 1.018–1.072) and a higher level of functional dependency at discharge (OR = 3.224 95% C.I. 1.558–6.542) were predictive for having more occupational gaps than the reference population 5 years after stroke (Table 3). Stroke severity was not found to be a significant predictor of occupational gaps. In this model 16.3%–21.8% of the variance was explained by the model.

**Table 3:** Predictive and explanatory regression models for having more occupational gaps than the reference population

	Wald	*p*‐value	Exp (B) (OR)	95% C.I. for OR
Predictive model				
Age at stroke onset	11.370	0.001	1.045	1.018–1.072
Functional dependency (mRS)	10.507	0.001	3.224	1.558–6.542
Stroke severity	0.250	0.617	1.223	0.556–2.691
Hosmer and Lemeshow	Chi‐2 df *p*‐value		8.790 8 0.360	
Cox & Snell	R2		0.163	
Nagelkerke	R2		0.218	
ROC‐curve	Area under curve *p*‐value		0.738 <0.001	
Explanatory model				
Age at time of follow‐up	12.375	<0.001	1.043	1.019–1.068
Feelings of depression	10.266	0.001	5.330	1.915–14.832
Hosmer and Lemeshow	Chi‐2 df *p*‐value		10.106 8 0.258	
Cox & Snell	R2		0.117	
Nagelkerke	R2		0.155	
ROC‐curve	Area under curve *p*‐value		0.711 <0.001	

mRS: modified Rankin Scale; OR: odds ration; CI: Confidence Interval; df: degrees of freedom; ROC: receiver operator characteristics.

The explanatory model showed that age at the time of follow‐up (OR 1.043 95% C.I. 1.019–1.068) and feelings of depression had a significant association with more occupational gaps than the reference population 5 years after stroke (OR = 5.330 95% C.I. 1.915–14.832). None of the variables excluded in the univariate analysis showed any significant contribution when inserted into the model. This model with these two variables explained 11.7%–15.5% of the variance in the outcome.

## 4 DISCUSSION

This study showed that 5 years poststroke, 50% of the respondents have higher number of gaps compared to age matched controls. The respondents’ age and level of functional dependency at stroke onset could predict the increased number of occupational gaps 5 years poststroke. Feelings of depression were an important explanatory factor. These results have important clinical relevance, as a higher number of gaps has been shown to contribute to reduced participation/health related quality of life (Eriksson, Aasnes, Tistad, Guidetti, & von Koch, 2012). These people need to be identified early after stroke in order to prevent reduced participation and isolation (Eriksson et al., 2012).

### 4.1 Occupational gaps in population

The present study showed that 71.1% of the participants experience occupational gaps 5 years after stroke and half of the study population experienced more gaps than the reference population. Previous studies have shown that approximately 90% experience occupational gaps 1 year (Bergstrom et al., 2012; Eriksson et al., 2012) or 5 years (Bergström et al., 2017) after stroke. An explanation for more experienced gaps could be the older study participants in two of the studies (Bergstrom et al., 2012; Eriksson et al., 2012) since an identified predictor for worse outcome after stroke is older age (Macciocchi, Diamond, Alves, & Mertz, 1998; Nakayama, Jorgensen, Raaschou, & Olsen, 1994) found in the present study as a predictor for an increased number of gaps 5 years after stroke. The activities *hobbies*,* participating in sports,* and *outdoor life* had the highest number of gaps in the present study. Previous studies have shown that *traveling for pleasure* (Bergstrom et al., 2012 *;* Eriksson et al., 2012) and *participating in sports* (Bergström et al., 2017) were the activities with most number of gaps. *Traveling for pleasure* had only the fourth highest number of gaps in the present study with a longer follow‐up time and with a younger study population, and only the seventh highest number of gaps in another 5 year follow‐up study conducted on a younger population (Bergström et al., 2017).

### 4.2 Predictors and explanatory factors

Age at the onset of stroke and level of functional dependency at discharge were identified as predictors for an increased number of occupational gaps 5 years after stroke. These variables could explain 16.3%–21.8% of the variance in the outcome, indicating existence of other important predictors. Few studies have investigated predictors for occupational gaps, but one study (Bergstrom et al., 2012) showed that stroke severity, activities of daily living abilities, social participation, and not being born in Sweden as predictors for occupational gaps, whereas age at stroke onset was not found to be a significant predictor. Furthermore, age and physical capability have previously been identified as predictors for a reduced subjective participation 2–4 (Desrosiers et al., 2006) and 6 (Singam et al., 2015) years after stroke, indicating generalizability of the identified predictors in the present study since the previous study populations were older and had different sex distribution compared to the present study.

The explanatory model showed that age at time of follow‐up and feelings of depression contributed to an increased number of occupational gaps 5 years after stroke. The present study found that 13.9% of included individuals had feelings of depression often or always, while 37.6% sometimes had feelings of depression. 17% reported taking medication for their feelings of depression, which is in line with the reported prevalence of depression which ranges from 19% to 33% (Hackett, Yapa, Parag, & Anderson, 2005; Robinson, 2003). No studies were found about association between depression and occupational gaps, but depression has been identified as a negative factor for participation after stroke (D'Alisa, Baudo, Mauro, & Miscio, 2005; Micaela Silva et al., 2016), which strengthens the findings of association between depression and occupational gaps after stroke.

### 4.3 Methodical considerations

There is a risk with questionnaires that the respondent misunderstand the questions and therefore answer incorrectly. Furthermore, a next of kin can have help the respondent to fill out the questionnaire, resulting in a risk of lower accuracy. Since the questions in the OGQ are answered with yes or no, a partial restriction in an activity will not be apparent in the questionnaire.

Ordinal and categorical variables were dichotomized primarily from a clinically relevant perspective and secondarily to attain satisfactory group sizes for logistic regression. The process of dichotomization comes with a loss of information (Fedorov, Mannino, & Zhang, 2009) and consequently a reduction in statistical power (Fedorov et al., 2009). Cut‐off levels for NIHSS used in the present study were chosen because of a high prevalence of milder stroke. These levels have been used previously (Muhr, Persson, & Sunnerhagen, 2017) but are not validated. The merging of NIHSS and H&H into a dichotomized variable for stroke severity is not validated and based solely on clinical experience; however, no conclusions were based on the results from this variable.

### 4.4 Study limitations

The participants were all from Sahlgrenska University Hospital, which is the sole hospital in the region that carries out thrombolysis, thrombectomy, and neurosurgical treatment. This result in younger patients being relocated to Sahlgrenska, lowering the mean age of the study population. The present study population's mean age was roughly a decade younger than the national average patient with stroke during 2009–2010 (The Swedish stroke registry, 2009, 2010). Furthermore, 13%–17% of patient with stroke admitted to Sahlgrenska University Hospital were not treated in a stroke unit or neurosurgical unit during the inclusion period (The Swedish stroke registry, 2009, 2010) and were therefore not included in the present study. Patients’ admittance to nonstroke units could be due to milder cases of strokes which would give the study population a higher representation of more severe stroke cases.

The percentage of respondents was 61.5%, which is equivalent to the response rate of most questionnaires sent out in Sweden (Jansson, Tipple, & Weissenbilder, 2017). The nonrespondents had significantly more women and more severe cases of stroke which might reduce the generalizability of the present study. Furthermore, the follow‐up in this study comprised five different questionnaires, resulting in a time consuming survey that could dissuade persons with lack of endurance or other limitations from participating, which might also reduce the generalizability of the present study.

### 4.5 Clinical implications

Results from the present study provide knowledge about participation and occupational gaps after stroke, as well as information regarding predictors and factors associated with occupational gaps.

The individuals’ feelings of participation are important to obtain a full view of their well‐being and to highlight needs for interventions.

The knowledge of predictors is important to be able to identify individuals at risk for reduced participation. Older stroke survivors and stroke survivors with an increased functional dependency could benefit from further evaluation and rehabilitation interventions to enable a more favorable outcome regarding participation in everyday occupations. Health care professionals considering depression in stroke survivors could benefit these individuals and lead to a higher degree of participation.

Further research is needed to evaluate the extent of occupational gaps in different populations and settings as well as to strengthen the knowledge on predictors for occupational gaps.

## CONFLICTS OF INTERESTS

The authors state that there are no conflicts of interests.
